# Signals and Cells Involved in Regulating Liver Regeneration

**DOI:** 10.3390/cells1041261

**Published:** 2012-12-13

**Authors:** Liang-I. Kang, Wendy M. Mars, George K. Michalopoulos

**Affiliations:** Department of Pathology, University of Pittsburgh, Pittsburgh, PA 15261, USA; E-Mails: lik19@pitt.edu (L.I.K.); wmars@pitt.edu (W.M.M.)

**Keywords:** liver regeneration, hepatocyte, hepatocyte growth factor, epidermal growth factor, partial hepatectomy, transdifferentitation, oval cell

## Abstract

Liver regeneration is a complex phenomenon aimed at maintaining a constant liver mass in the event of injury resulting in loss of hepatic parenchyma. Partial hepatectomy is followed by a series of events involving multiple signaling pathways controlled by mitogenic growth factors (HGF, EGF) and their receptors (MET and EGFR). In addition multiple cytokines and other signaling molecules contribute to the orchestration of a signal which drives hepatocytes into DNA synthesis. The other cell types of the liver receive and transmit to hepatocytes complex signals so that, in the end of the regenerative process, complete hepatic tissue is assembled and regeneration is terminated at the proper time and at the right liver size. If hepatocytes fail to participate in this process, the biliary compartment is mobilized to generate populations of progenitor cells which transdifferentiate into hepatocytes and restore liver size.

## 1. Introduction

### 1.1. Overview

The liver is a multi-functional organ that controls key physiological processes. These include nutrient processing following intestinal absorption, waste processing and excretion (urea cycle and bile synthesis), detoxification of xenobiotics, energy and nutrient storage and regulation, production of serum proteins (coagulation factors, oncotic proteins, carrier proteins) and hormones (thrombopoietin [1], IGF1), as well as other functions.

The ability of the liver to carry out these normal duties is so essential that liver mass is maintained within a very narrow range in relation to the overall body mass. If there is loss or gain of liver mass, such as through liver injury or pregnancy, respectively, compensatory proliferation or apoptosis of cells allow restoration of original liver/body mass ratio once the stimulus is removed. The term “hepatostat” has been coined to describe this unique homeostatic relationship [2]. When the hepatostat is derailed and loss of liver function due to parenchymal injury falls below a critical point (build-up of toxic metabolites, inability to maintain glucose levels and blood pressure, along with coagulopathy), multi-organ failure and death follow.

The robust programmed proliferative response to loss of parenchymal function is widely known as “liver regeneration”. A more accurate description may be “compensatory hyperplasia and hypertrophy”, as resection of the liver does not induce spatial replacement of the part of the organ that was lost. Instead, the cells in the remaining portion proliferate and/or increase in size to restore the original liver mass [3]. Repopulation of the liver can be achieved via one of two mechanisms: (1) self-replication of individual cell types, or (2) transdifferentiation from facultative stem cells, or liver progenitor cells (LPC). In this review, we use the term “liver regeneration” to refer to restoration of the liver parenchyma by either of these processes.

Management of chronic liver injury and its sequelae are growing health care burdens worldwide [4]; knowledge of the principles and cellular compartments governing successful restoration of liver function after insult is the key to discovering therapeutic strategies applicable to human hepatic disease. This review highlights the molecular and cellular inputs necessary to complete liver regeneration after loss of parenchyma.

### 1.2. Cell Types of the Liver

**Figure 1:**
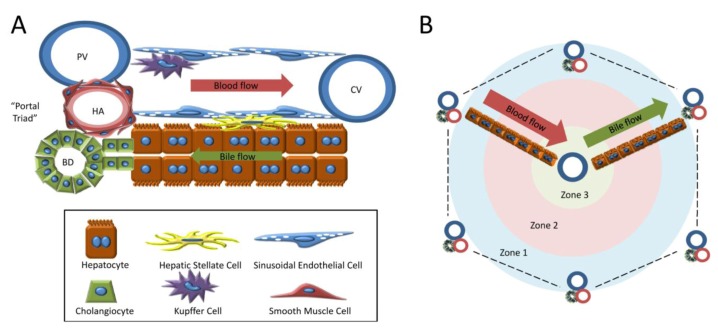
Architecture of the liver.** (a) **Schematic depicting the architecture and cell type composition of the hepatic sinusoid. Blood enters the lobule from branches of the portal vein (PV) and hepatic artery (HA) and progresses through the sinusoidal capillaries, collecting at the central veins (CV). Bile flows in the opposite direction towards the portal triad and exits through the bile ducts (BD). Hepatocytes are lined up along the sinusoids in “plates” 1-2 hepatocytes thick. Hepatic stellate cells reside in between the sinusoidal endothelial cells and hepatocytes in the “space of Disse”. **(b) **Diagram illustrating the organization of the hepatic lobule, including vascular structures and relative zonality of the lobule. This unit is repeated throughout the liver. Representative plates of hepatocytes are shown for orientation; these rows of hepatocytes would fill the entire lobule and be lined with sinusoids.

The main unique cell types of the liver are hepatocytes, cholangiocytes (or biliary epithelial cells), Kupffer cells, hepatic stellate cells, and sinusoidal endothelial cells. Hepatocytes are organized into cords that line an intricate, specialized capillary bed lined with fenestrated endothelial cells (Figure 1a). The vascular network is organized into a system that allows for unidirectional flow from branches of the inflow vascular supply, the portal vein and the hepatic artery, through the sinusoids to the central veins, which coalesce into the hepatic vein and connect to the inferior vena cava. The portal vein and hepatic artery are found in cluster along with a collecting bile duct, collectively referred to as the “portal triad” and spaced out at the corners of a roughly hexagonal unit (“the hepatic lobule”, Figure 1b) that is repeated throughout the liver tissue. The lobule can be divided into three general zones: periportal (zone 1), pericentral (zone 3), and transitional (zone 2). The hepatocytes in Zone 1 to Zone 3 are exposed to increasing concentrations of processed xenobiotics/toxins and decreasing concentrations of oxygen (Figure 1b).

The hepatocyte is the parenchymal cell of the liver, performing all the essential functions of the organ described in the section above. In addition, the hepatocytes produce bile, secreted from the apical membrane into the bile canaliculi that run between hepatocytes and merge to form bile ducts, which are lined with cholangiocytes. Cholangiocytes and hepatocytes share a common precursor cell, the hepatoblast, in development. This common lineage is attributed for the ability of cholangiocytes and hepatocytes to transdifferentiate in the setting of injury where one or the other cannot replicate sufficiently to replace its own cell compartment. The “non-parenchymal cell” population includes sinusoidal endothelial cells, Kupffer cells, and hepatic stellate cells. Sinusoidal endothelial cells are fenestrated to allow passage of macromolecules and lipoproteins in and out of the hepatocytes. Kupffer cells are resident liver macrophages, which along with other liver-associated immune cells play important roles in immune tolerance of the liver [5]. The hepatic stellate cell is a multifunctional cell of mesenchymal origin found to have roles in immune function, vitamin storage, matrix turnover, growth factor secretion, vascular tone, and perhaps progenitor cell niche. During injury, hepatic stellate cells become “activated” and transform into myofibroblast-like cells, depositing extracellular matrix and becoming contractile [6]. 

During liver regeneration, whether following surgical resection or parenchymal injury and loss, all of the cell types of the liver can proliferate to replace their own cell population. When unable to do so, however, there are internal or external (e.g., hematopoietic) sources of progenitor cells that are able to differentiate into the various cells of the liver to restore cell compartments. These processes will be discussed in more detail below.

### 1.3. Models of Liver Regeneration and Growth

The two-thirds partial hepatectomy (PHx) model was first described in 1931 [7] and remains one of the most widely used models of liver regeneration. The rodent liver is multi-lobular, the two largest of which approximates 70% parenchymal mass. When these two lobes are excised via a straightforward surgical procedure [8], the cells of the remaining lobes restore liver mass over the course of 1-2 weeks. There are two key advantages to this approach: 1) the model is easily scalable, allowing investigators to study phenomena associated with minor (~30% PHx) to severe (~90% PHx) parenchymal loss simply by removing one less or one more lobe of the liver, and 2) because there is no injury to the remaining hepatocytes, PHx provides a “clean” model in which to study the timing and extent of contribution of different variables, which has been less optimally studied in injury models using hepatotoxins that are associated with necrosis/inflammation. However, the main drawback to this approach is the limited applicability of the PHx model for interpreting the dynamics of regeneration in human disease, which often involves components of hepatocyte death and inflammation. In addition, PHx alone does not require LPCs for successful regeneration, and thus is not a useful model to study LPC-mediated regeneration.

In comparison, several chemical injury models also exist. These cause hepatocyte injury and death, which activates an inflammatory response in addition to a regenerative response. One commonly used class of agents causes hepatocyte injury and death selectively in the pericentral zone (Zone 3); these include carbon tetrachloride [9,10] and acetaminophen [11,12,13]. They require metabolic activation by cytochrome P450 enzymes, a process which often generates hepatocyte-toxic free radicals. The CYP-expressing hepatocytes die first, creating a centrilobular distribution of injury and death. Allyl alcohol administration has been used to induce acute periportal injury [14]. There are two main advantages in using a hepatotoxin model to study regenerative response after parenchymal injury: (1) it more closely approximates the regenerative response that occurs in common human hepatic diseases, including the damage and inflammatory infiltrate, and (2) unlike PHx [15], there are few surgical complications with repeated administrations, so it is easy to extend the acute injury model to chronic injury and cirrhosis. An emerging concept from the hepatic toxicology field is the “progression of injury”: primary injured/necrotic hepatocytes release phospholipases [16,17] and proteases [18] into the extracellular space, which then injure neighboring hepatocytes and delay/inhibit their ability to undergo normal regeneration. It is likely these processes occur in a variety of acute and chronic human liver diseases and intoxications, where there is also *in situ* hepatocyte injury and death. Therefore, despite the relative lack of clarity about cellular origins of signals occurring as a result of chemical injury models, the importance of synthesizing our understanding of ideal liver regeneration from PHx with data from chemical injury models cannot be understated. It may be the key to future understanding of the limitations of current interventions and to finding more suitable therapeutic targets for human disease and acute intoxication. These topics, however, are outside the scope of this review, and the reader is referred to other reviews discussing hepatic injury progression in chemical intoxication models for more detail [19,20]. 

A final model of liver growth is augmentative hepatomegaly (also known as “direct hyperplasia”), in which liver is stimulated to grow to a supraphysiological mass by growth factors, hormones [21,22,23,24], or xenobiotics. There are two classes of xenobiotics commonly used in this experimental model: peroxisome proliferator-activated receptor (PPAR) family agonists [25] and *constitutive androstane receptor* (CAR) agonists [26]. Continuous administration of these chemicals induces liver growth until a new equilibrium is reached, which is different for each chemical. Upon removal of the hormone or xenobiotic treatment, however, the liver shrinks back to the original mass through hepatocyte apoptosis [27,28,29]. These phenomena suggest that the innate hepatostat is disrupted or readjusted in response to these xenobiotics, returning to normal when the chemical is removed, although it is not clear which pathways are relevant for this purpose. Due to the dependence on specific nuclear receptor pathways, there are some key differences between augmentative hepatomegaly and normal liver regeneration (compensatory hyperplasia) [30]. However, it has been shown that the same genetic alterations that enhance [31] or suppress [32] compensatory hyperplasia can also enhance [33,34] or suppress [35] augmentative hepatomegaly, so lessons learned from the augmentative hepatomegaly model may bear relevance to enhancing compensatory liver regeneration. There are scenarios where augmentative hepatomegaly is clinically relevant: (1) elevated estrogens during pregnancy are thought to increase liver weight to meet increased metabolic needs, (2) several prescribed drugs, such as phenobarbital [36], phenytoin, and diazepam, can bind to CAR and sensitize the patient to acetaminophen toxicity.

For consistency in interpretation, the signals and cellular dynamics to be covered in the main portion of this review focus on the extensive body of knowledge amassed from PHx studies in rodents.

## 2. Molecular Signals During Liver Regeneration after PHx

What initiates liver regeneration after PHx? Immediately upon removal of two-thirds of the liver in the standard PHx model, the entire hepatic vascular influx is forced to perfuse through only one-third of the original capillary bed. Consequently, there is an increase in portal and capillary pressure, as well as an increased availability of circulating growth factors and hormones. While studies are lacking in how the mechanical forces can directly influence gene expression during liver regeneration after PHx, there have been many observations that early changes in circulating factors, cell-cell and cell-matrix interaction, as well as intracellular signaling cascades dictate the kinetics of regeneration.

If portal circulation is maintained at normal pressures after PHx in rats using a portacaval shunt, there is an absence of hepatocyte hypertrophy and decreased protection against apoptosis compared to control PHx; these effects are attributed to lack of hepatocyte growth factor (HGF) activation [37]. Indeed, even the tonic maintenance of the hepatostat may be regulated in part by availability of circulating factors; in the absence of PHx, complete shunt of portal flow to the vena cava induces liver atrophy to one half of the original size [38]. In this portacaval shunt model, exogenously infused growth factors such as insulin, transforming growth factor-α (TGF-α), and HGF exhibited direct hepatotrophic effects, highlighting the importance of circulating growth factors in initiating hepatocyte growth and proliferation after a stimulus as well as maintenance of baseline liver size.

Extracellular matrix reorganization is another early feature after PHx that has profound effects on initiation of liver regeneration by releasing locally available latent growth factors. Urokinase-type plasminogen activator (uPA) activity increases within one minute of PHx, initiating a matrix remodeling cascade via plasminogen activation (within 15 minutes of PHx), which activates key metalloproteinases (MMP) such as MMP-9 [39,40]. Importantly, uPA activates HGF in a manner utilizing its receptor uPAR, which starts increasing within one minute of PHx [41,42,43]. 

Changes in cell junction proteins and related signaling are also early features of regeneration. One example is the transmembrane protein Notch. Its intracellular domain, cleaved when Notch is activated by transmembrane ligands existing on neighboring cells, migrates to the nucleus and initiates transcription within 15 minutes of PHx [44]. Another example is β-catenin, a protein that promotes cell-cycle progression by complexing with the Tcf transcription factor family to activate target genes. β-catenin translocates to the nucleus within 5 minutes of PHx and remains elevated in the nucleus over 24 hours [45,46]. 

The early events described above are illustrative of the broad changes that occur in the liver after PHx. These and innumerable other factors have been examined in various models of liver regeneration, raising the question of whether all factors found in reductionist animal studies actually play physiologically-relevant roles in liver regeneration. Loss of even the most potent growth factors (HGF, EGFR ligands) does not completely arrest regeneration; instead, delays of various lengths are seen to complete regeneration. High-throughput studies have been designed to address this question [47,48,49] and literature reviews have synthesized current knowledge into schemas to better understand the redundancy that occurs between metabolic, growth factor, and cytokine effects [50,51]. Below, we will explore in more depth a select cohort of intra- and extracellular signals shown to play roles in liver regeneration using the PHx model. 

### 2.1. Hepatocyte Growth Factor (HGF)

HGF is one of only two described “complete mitogens” for hepatocytes (defined by its ability to induce DNA synthesis of primary hepatocytes in a chemically defined serum-free medium *in vitro* and liver enlargement when administered *in vivo*). It was one of the first isolated and studied circulating factors found to promote liver regeneration [52,53,54,55]. HGF binds to and activates the tyrosine receptor kinase MET, a multifunctional receptor involved in a number of cellular processes, such as proliferation, growth, survival, and metabolism [56,57,58]. Decrease of MET expression and activation using RNA interference (shRNA) is sufficient to inhibit mitoses and increase apoptosis related genes at 24 hours post-PHx, normally the peak of proliferation in the rat [59]. The mitogenic activity of HGF-initiated signaling requires the function of the transcription factor C/EBPβ [60].

HGF is utilized during liver regeneration after PHx in a biphasic manner. In the resting liver, HGF (inactive) is stored in the extracellular matrix of the liver, particularly in the connective tissue surrounding portal triads [61,62]. After PHx, the first wave of MET activation occurs within 30 minutes and peaks at 60 minutes [63]. This first round of HGF/MET signaling is thought to be derived from endogenously present HGF, as levels of both inactive and active HGF in the liver tissue decrease from baseline levels during the first three hours after PHx [64]. Early extracellular matrix remodeling events in the liver [39] allow the matrix-bound HGF to be activated and utilized, and some is released into the circulation as well [65]. The second phase of HGF utilization, this time from newly-synthesized HGF by stellate cells and endothelial cells [66,67,68], begins at three hours post-PHx and peaks at 24 hours after PHx [64,69]. 

Extrahepatic sources of HGF, such as platelets [53] and other organs (lung, kidney, spleen) [70,71], may also contribute to the pool of available HGF during liver regeneration, although the relative importance of these sources is not known. Systemic induction of HGF expression after PHx by different organs, including liver, may be a response to increases in circulating norepinephrine [72] or insulin-like growth factor [73], which have been shown to stimulate HGF mRNA transcription.

### 2.2. Epidermal Growth Factor Receptor Ligands

Ligands of the epidermal growth factor receptor (EGFR, or ErbB1) comprise the only other “complete mitogens” known besides HGF. Although there are other known ligands, the EGFR ligands studied in the context of liver regeneration to date are as follows: epidermal growth factor (EGF), transforming growth factor-alpha (TGF-α), amphiregulin, and heparin binding-EGF-like growth factor (HB-EGF). TGF-α, amphiregulin, and HB-EGF are synthesized as transmembrane-anchored precursors that are then processed by extracellular proteases to their mature secreted forms.

EGF is mitogenic to hepatocytes, both in culture [74] and in the resting liver in rats when infused exogenously [75]. Although EGF is produced by multiple tissues, the two shown most relevant to liver regeneration include the Brunner’s glands in the duodenum and salivary glands. The Brunner’s glands provide a tonic supply of EGF via the portal circulation to the liver, where it is taken up and sequestered in the portal triad areas [76,77]. Increased circulating norephinephrine, as is seen after PHx, can augment Brunner’s glands secretion of EGF [78]. Sialadenectomized rats exhibit severely blunted liver regeneration post-PHx, which can be rescued by exogenous EGF [79]. 

TGF-α is a more potent hepatocyte mitogen than EGF; this is thought to be mediated through differential ligand-receptor complex processing [80]. It is produced by hepatocytes during regeneration [81] and cleaved by specific proteases into the active growth factor [82,83]. In this way, it perhaps functions in an autocrine [84] or paracrine manner to contribute to liver regeneration. However, TGF-α null mice have no deficiency in liver regeneration [85].

Similar to TGF-α, amphiregulin is also produced by hepatocytes after PHx, and in culture its expression is regulated by interleukin-1β (IL-1β) and prostaglandin E2 [86,87]. Interestingly, the same study reports that amphiregulin knockout mice have impaired liver regeneration post-PHx, in contrast to TGF-α knockout mice.

HB-EGF is synthesized by Kupffer cells and endothelial cells during liver regeneration [88]. It has been found to be a potent mitogen for rat hepatocytes in culture [89]. In mice, HB-EGF is expressed in two-thirds PHx (where there is significant hepatocyte proliferation) but not one-third PHx (where there is minimal hepatocyte proliferation). However, exogenous HB-EGF given after one-third PHx is able to induce DNA replication in hepatocytes to comparable levels as with the two-thirds PHx at 24 hours [90]. HB-EGF transgenic mice have accelerated liver regeneration [91], and mice lacking HB-EGF have delayed regeneration [90].

Activation of the EGFR, as denoted by tyrosine phosphorylation, is present at baseline due to the steady flow of EGF from the Brunner’s glands, as discussed above. After PHx, though, activation increases above baseline with similar kinetics as MET activation; peak EGFR activation is observed at 60 minutes post-PHx [63]. Although other ErbB family receptors can be upregulated in compensation, loss of EGFR through shRNA interference in rats decreases DNA replication post-PHx [92], and hepatocyte targeted gene deletion in mice have higher rates of mortality post-PHx [93].

### 2.3. Tumor Necrosis Factor (TNF)

TNF is a pro-inflammatory cytokine produced by macrophages that has been shown to have pleiotropic roles during liver regeneration. On one hand, TNF mediates hepatocyte apoptosis and liver failure in mice in a number of toxicity models [94,95,96,97,98,99]; many of these studies also demonstrate a protective effect of NF-κB activation against the cytotoxic effects of TNF. On the other hand, a number of studies have demonstrated that loss of TNF function delays liver regeneration, whether by using TNF neutralizing antibodies [100], or TNF receptor 1 deficient mice [101]. It is likely that TNF signaling on cells already “primed” to survive and enter into proliferation can promote and enhance the same pathways, such as Akt and NF-κB activation [96,102] in response to growth factors [103,104]. In the absence of such signals, TNF then promotes death pathways.

### 2.4. Interleukin-6 (IL-6)

IL-6 is a multifunctional cytokine involved in initiating and mediating the acute phase response by hepatocytes during inflammation and other homeostatic disturbances [105,106,107]. It is secreted by immune cells, non-parenchymal cells, and hepatocytes under certain conditions [105] and signals through the IL6R-gp130 complex [108,109]. Plasma concentration of IL-6 increases after PHx, and mice lacking IL-6 display delayed liver regeneration and liver injury associated with loss of STAT3 activation and decreased cell cycle progression proteins such as Cyclin D1 [110]. A single dose of IL-6 prior to PHx could rescue the IL-6 deficient mice from liver damage and restore near normal regenerative capacity. Despite this marked phenotype, IL-6 alone is not mitogenic to hepatocytes in culture and administration of IL-6 to normal animals does not induce hepatocyte proliferation.

One potentially important role of IL-6 in liver regeneration, not fully explored, is its regulation of HGF. Recent studies suggest a feedback loop exists between IL-6 and HGF. The HGF gene promoter contains IL-6 response elements [111] and HGF has been shown to be synthesized in response to IL-6 by non-parenchymal cells of the liver [112,113]. HGF signaling through MET prevents NF-κB nuclear accumulation and decreases IL-6 synthesis in a GSK3β-dependent manner in bone marrow-derived macrophages [114], showing that HGF, in addition to its mitogenic and anti-apoptotic roles, may be a key anti-inflammatory mediator in liver repair [115].

### 2.5. Transforming Growth Factor-β (TGF-β)

These growth factor/cytokines, a subgroup of a larger TGF superfamily of ligands, include TGF-β1, 2, and 3, and their receptors are the TGF-β receptors type I, II, and III. Ligand binding to the type II receptor promotes complex formation with the type I receptor to initiate signaling as a heterodimer receptor complex [116]. The majority of studies in liver regeneration have focused on signaling mediated by TGF-β1. 

Hepatocytes express all three TGF-β receptors [117,118]. It has been shown that locally, all the non-parenchymal cell types are capable of expressing TGF-β1, with Kupffer cells and endothelial cells expressing the most at baseline, and hepatic stellate cells being the major cellular source during inflammatory and fibrogenic conditions [119,120]. There are conflicting reports about whether hepatocytes can express TGF-β1 during regeneration [119,121,122]. HGF and EGF-induced signaling can promote TGF-β expression in organoid cultures [123]. Hepatocytes are exquisitely sensitive to the mitoinhibitory effects of TGF-β1 in culture [124,125] and* in vivo* [126]. 

At baseline, extracellular but latent TGF-β1 can be detected throughout the lobule of the liver [127]. After PHx, matrix-bound TGF-β1 becomes activated and released into the circulation; increases in concentration are detected within one hour of PHx [128]. A strong “wave” of mature TGF-β1 immunolocalization can be observed in the initial stages of regeneration after PHx (12-48 hours), progressing in a periportal to pericentral direction. Likewise, a wave of hepatocyte proliferation is observed to follow immediately adjacent to this line, in TGF-β1-negative regions [127]. Despite the large increase in TGF-β1 activation, hepatocytes isolated between 24-72 hours after PHx are resistant to the mitoinhibitory effects of TGF-β1 in culture [129]. This may be due to a combination of downregulated TGF-β receptor expression [118] and inactivation of mature TGF-β1 by α2-macroglobulin in the circulation [130]. TGF-β1 mRNA levels increase by 4 hours after PHx and reach maximal expression by 72 hours [131]. Genetic deletion of the TGF-β type II receptor in hepatocytes enhances hepatocyte proliferation after PHx in mice but does not prolong regeneration [132] (see section 4 below for more discussion on TGF-β in termination of regeneration). Loss of the *β*-2 spectrin protein, an adaptor protein involved in the TGF-β signaling pathway, also increases markers of proliferation at 24 hours; however, by 48 hours, those markers were decreased coincident to elevations in markers of cell cycle arrest (p53, p21) and DNA damage [133]. Consequently, the pleiotropic roles of TGF-β1 in liver regeneration are not fully understood.

### 2.6. Neurotransmitters

Norepinephrine is produced by cells of the sympathetic nervous system, where it functions as a neurotransmitter, and by the adrenal medulla, from which it is secreted into the circulation as a stress hormone. Furthermore, hepatic stellate cells have been observed to produce norepinephrine [134]. Norepinephrine concentrations increase in the liver after PHx with similar kinetics as HGF [135]. Although it lacks mitogenicity in itself, norepinephrine stimulates the production and potentiates the mitogenic effects of HGF [65,72] and EGF [78,136,137], and it weakens the mitoinhibitory effects of TGF-β [125,129]. These effects are attributed to SMAD7 [138], NF-κB [138], and STAT3 [139] activation by norepinephrine. Chemical inhibitors of α-1 adrenergic receptors delay liver regeneration [135].

Serotonin is another neurotransmitter with noted effects on liver regeneration. Although not produced by platelets, it is stored and secreted by them to regulate hemostasis and inflammatory processes. Mice lacking platelets or tryptophan hydroxylase 1 (the rate-limiting enzyme for peripheral serotonin synthesis) have deficient proliferative response at 48 hours after PHx, which can be rescued by exogenous serotonin administration [140]. The same group reports that a serotonin receptor agonist can promote sinusoidal endothelial cell fenestration and increased hepatocyte proliferative response after PHx in a VEGF-dependent manner [141]. Since VEGF stimulates HGF production, it is therefore possible that serotonin indirectly promotes hepatocyte proliferation through increasing HGF levels, although this has not been measured. Indeed, the exact mechanisms of serotonin action are not well understood. Rats lacking the serotonin transporter (SERT) are unable to store serotonin in their platelets and blood serotonin concentrations are only 1–6% of normal levels; paradoxically, they exhibit no proliferative defect after PHx [142]. It may be possible that even trace amounts of serotonin in the SERT -/- rats are sufficient to exert its effects after PHx, or there may be inherent differences in serotonin sensitivity between the mouse and the rat.

### 2.7. Bile Acids

Hepatocyte proliferation has been long noted as a histologic feature of human cholestatic conditions. Now there is increasing evidence that bile acids promote hepatocyte proliferation during regeneration. The concentration of bile increases in the blood within several hours after PHx, and depletion of bile or deficiency in the bile-responsive transcription factor FXR delays regeneration [143]. Bile acids also activate the receptors LXR [144] and TGR5 [145] to increase resistance against bile acid toxicity in hepatocytes and cholangiocytes, respectively.

### 2.8. Insulin and Other Metabolic Regulators

The liver processes and stores the three macronutrients (carbohydrate, protein, and lipid). Altered regulation of macronutrients is tightly orchestrated during liver regeneration to promote successful DNA synthesis and cell replication, and disruption of these compensatory mechanisms leads to impaired regeneration [146,147]. 

Insulin is a key regulatory hormone for liver homeostasis and function. Liver is the first target organ for freshly secreted insulin from the beta islet cells in the pancreas. Without insulin, primary hepatocytes cannot survive in culture [148] and are unable to respond to mitogens [137,149]. In the absence of PHx, portacaval shunt induces liver atrophy to about one-third of the original size; injection of insulin in this model is able to restore liver mass through hepatocyte proliferation [38]. However, insulin alone does not induce hepatocyte proliferation or growth of liver [150]. Further, after PHx, rodents become hypoglycemic and hypoinsulinemic; external supplementation of dextrose suppresses hepatocellular proliferation [151,152]. It is therefore likely that while basal insulin is important for normal hepatocyte homeostasis, insulin action during hepatic insufficiency counteracts the metabolic stimuli necessary to initiate hepatic regeneration (such as lipolysis, described below). 

The availability of certain amino acids is a rate-limiting step for hepatic regeneration. Protein deprivation significantly delays DNA synthesis after PHx [153], while supplementation with branched chain amino acids (BCAA) promotes liver regeneration [154]. Interestingly, other amino acids have no effect or inhibit regeneration. The specific effects of BCAA supplementation can be attributed to stimulation of protein and nucleotide synthesis [146].

 Systemic lipolysis and hepatic lipid accumulation (steatosis) are early cardinal features of liver regeneration [155,156]. In addition, DNA synthesis after PHx is enhanced by exogenous administration of long chain fatty acids or L-carnitine (an essential co-factor for transport of acyl groups into the mitochondria for β-oxidation) [157]. It is thought that the suppressive effects of dextrose on regeneration are mediated indirectly through suppression of peripheral free fatty acid release by insulin [158]. However, the exact functions of hepatic steatosis (energy source? signaling?) after PHx are not well elucidated [147].

### 2.9. Wnt Family Proteins/β-catenin

As mentioned above, β-catenin nuclear translocation is one of the earliest events after PHx. β-catenin is normally sequestered in complex with E-cadherin at tight junctions of hepatocytes or tagged for ubiquitination and degradation; however when Wnt family proteins become available after PHx and activate the frizzled receptors, β-catenin is stabilized and can accumulate in the nucleus [45]. Wnt ligands and frizzled receptors are expressed by various liver cell types, both parenchymal and non-parenchymal cells [159]. Tyrosine receptor kinases such as MET have also been found to promote β-catenin stabilization and translocation to the nucleus [160,161]. β-catenin gene knockdown in rats [162] or liver-specific conditional knockout in mice [163] suppresses proliferation after PHx and delays regeneration.

### 2.10. Hedgehog

Hedgehog (HH) ligands bind to the receptor Patched, dissociating it from Smoothened and allowing Smoothened to initiate intracellular signaling. Both Indian and Sonic HH are expressed in the liver by various cell types [164,165], although it is not clear which cells express Patched. Inhibition of HH signaling using cyclopamine in mice caused dramatic inhibition of cell proliferation at 48 hours post-PHx and almost 100% mortality by 72 hours, despite no difference in serum liver injury markers at 24 hours and 48 hours post-PHx. In addition, activation of hepatic stellate cells and bile duct proliferation was also suppressed [166]. Further work is needed to elucidate the mechanisms behind these results, but they reveal HH signaling to be a major driving force behind liver regeneration. Modulation of this pathway, such as inhibition by glypican-3 [167], could therefore be a molecular switch to terminate liver regeneration (see below for more discussion on glypican 3).

### 2.11. Fibroblast Growth Factors (FGFs)

Fibroblast growth factors (FGF) are a large family of proteins (23 known members and counting), for which there are four known receptors (FGFR1-4). All four are expressed by different cell types in the liver, but hepatocytes express FGFR4 exclusively [168]. FGF1, FGF2 and FGF7/KGF can stimulate moderate DNA synthesis in cultured hepatocytes [169,170]. Disruption of all FGFR signaling through expression of a dominant negative receptor in hepatocytes resulted in reduced proliferation at 48 hours post-PHx [171], highlighting a role for parenchymal FGF signaling in regeneration. 

## 3. Contribution of cell types to successful liver regeneration

### 3.1. Resident Liver Cells

After PHx, a well-orchestrated set of cell replications occurs amongst the resident cells of the liver in order to replace the lost liver mass and function. First and foremost, the hepatocytes undergo cell proliferation, with the peak of proliferation being at approximately 24 hours in the rat [172] and approximately 36–42 hours in the mouse [8]. Cholangiocytes respond to the same mitogenic signals and start proliferating almost as early as hepatocytes, while non-parenchymal cells initiate DNA synthesis at a slower pace, with Kupffer cells and stellate cells peaking at 48–72 hours and sinusoidal endothelial cells at 96 hours [172,173,174]. There is no evidence to suggest that there are increased numbers of portal triads or lobules after completion of regeneration, and consequently, each lobule increases in size. 

Most hepatocytes participate in cell proliferation during regeneration to restore liver mass. In the two-thirds PHx model in younger rats, over 95% of hepatocytes undergo DNA synthesis, and this only decreases to about 75% even in older rats [175,176,177]. Exogenous infusion of mitogens can improve the proliferative response in older animals, indicating that age-related decreases in hepatocyte proliferation are not due to inherent inability to proliferate (senescence) but perhaps changes in the ability to respond to extracellular environment and signals [22,178]. The remarkable capacity of hepatocytes to undergo innumerable proliferation cycles is demonstrated by successful regeneration after serial PHx (up to 12 documented [179]) and serial repopulation of small numbers of isolated hepatocytes into host animal livers with impaired native cells (calculated 69 doublings of each cell over six repopulations [180]). Indeed, during regeneration hepatocytes express markers of stem cells, or reprogramming factors, such as Oct4, Nanog, and KLF4 [181], lending even more credence to their self-renewal capabilities.

**Figure 2:**
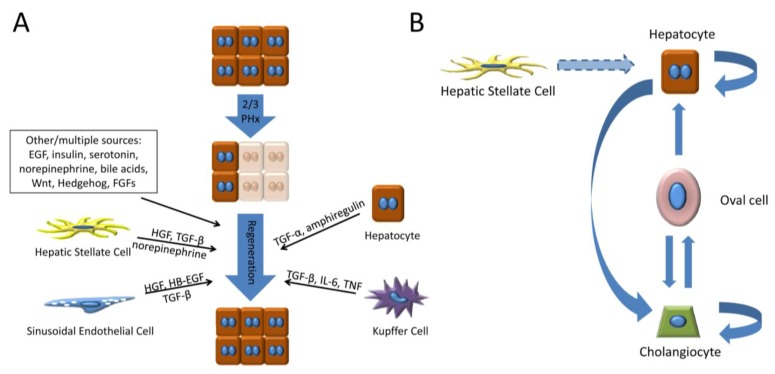
Signals and cells contributing to cell repopulation during liver regeneration.** (a) **Upon loss of two-thirds of the liver mass after PHx, molecular signals from several cell types contribute to hepatocyte repopulation during regeneration. **(b) **Both hepatocytes and cholangiocytes undergo self-renewal in normal regeneration; however, transdifferentiation can occur via the relationships indicated. Dotted arrows indicate relationships based on newly emerging literature.

During the regenerative process, hepatocytes can secrete many growth factors to which non-parenchymal cells are responsive. These include TGF-α [84], FGFs [182], VEGF [67], and PDGF-A [183]. In turn, the non-parenchymal cells provide to hepatocytes many of the growth factors discussed in detail earlier in this review. New HGF is synthesized by stellate cells and endothelial cells [66,67,68]. EGF production increases from Brunner’s glands in the duodenum, and HB-EGF is available from endothelial cells and macrophages [89]. Macrophages provide IL-6 and TNF-α. The secretion of growth factors by the different cell types to regulate hepatocyte proliferation during regeneration is summarized in Figure 2a.

Revascularization of the hepatic plates after proliferation occurs through a signaling “conversation” between hepatocytes and endothelial cells. Increases in expression of growth factor receptors on endothelial cells during regeneration, such as VEGF receptors Flk-1/KDR and Flt-1, allow them to be responsive to hepatocyte-derived VEGF [184]. The endothelial cells that surround avascular clumps of newly-replicated hepatocytes also selectively express the Angiopoietin receptor Tie-1 [184]. Activation of these VEGF and Angiopoietin receptors induces the endothelial cells to proliferate and invade in between the hepatocytes to form new sinusoids [173,185,186].

### 3.2. Progenitor Cells

#### 3.2.1. Bone Marrow Derived Cells

Mobilization of bone marrow has been reported after PHx in rodents [187] and also in humans [188]. Circulating bone marrow-derived mesenchymal stem cells contribute to new sinusoidal endothelial cell repopulation and are a major source of newly synthesized HGF after PHx [189,190]. It has been a topic of discussion whether circulating bone marrow cells contribute to hepatocyte repopulation during regeneration. In experimental models in which hepatocyte proliferation was inhibited, hematopoietic lineage cells were able to repopulate a portion of the hepatocytes [191,192]. However, more recent studies have suggested that the observations made from those experiments were in fact due to cell fusion events between the bone marrow hematopoietic stem cells and hepatocytes [193,194].

#### 3.2.2. Oval/Progenitor Cells and Transdifferentiation

While there is still an ongoing search for conclusive proof of a resident liver stem cell population, a variety of studies have established that several liver cell types can act as facultative stem cells and transdifferentiate to replace the epithelial cell compartments during regeneration [195,196]. 

One of the best-described examples is the emergence of “oval cells” in experimental models (e.g., administration of the chemical AAF prior to PHx) and diseases where hepatocytes cannot proliferate [197,198,199]. Oval cells are named based on the shape of their nuclei, and they express cell markers of both hepatocytes and biliary cells as well as stem cell markers [200]; they can be induced to differentiate into either cell type (Figure 2b). They appear in the periportal areas, and pulse-chase labeling of these cells with tritiated thymidine in the AAF + PHx model indicate that over the course of regeneration, they acquire hepatocyte-associated markers and phenotypic characteristics [197]. Many of the growth factors discussed earlier in this review have been implicated in oval cell differentiation into hepatocytes, such as TGF-α, HGF, TGF-β, and Notch [201,202,203,204], although some oval-cell specific signaling pathways have been identified [205,206]. Some disagreement exists about the origins of oval cells; however, it is likely that they derive from the biliary cell compartment for the following reasons: (1) most “oval cell markers” are shared markers with biliary cells (cholangiocytes), (2) in situations where they appear, such as massive hepatocyte necrosis of the human liver, they emanate from and cluster around the portal tract [207], (3) early in oval cell-inducing experimental protocols, hepatocyte markers (e.g., HNF4) appear in the nuclei of biliary cells of intact bile ducts prior to the appearance of oval cells, which also express these hepatocyte markers [208], and (4) toxin-mediated damage to the bile ducts prior to the AAF + PHx protocol can completely prevent the appearance of oval cells [209]. A proliferative biliary response, termed “ductular reaction”, is seen in many human liver disease conditions in which there is attrition of hepatocytes, it is likely that oval cell-mediated regeneration is relevant to human liver regeneration [210].

Hepatocytes have also been noted to possess transdifferentiation capabilities under certain conditions, particularly hepatocytes immediately proximal to the portal tract [195]. In experiments where hepatocytes positive for the DPPIV marker were injected into DPPIV-negative rats that had been subjected to PHx and retrorsine intoxication, the regenerated chimeric liver possessed no DPPIV-positive bile ducts unless a biliary proliferative stimulus (e.g., bile duct ligation) also accompanied the procedure. In the combined hepatocyte injury and biliary proliferation scenario, approximately 1.5% of the bile ducts became DPPIV-positive. If the ability of the native cholangiocytes to proliferate was inhibited by the biliary-specific toxin methylene dianiline (DAPM), the number of DPPIV-positive bile ducts increased to nearly 50% after bile duct ligation + PHx/retrorsine [211], demonstrating the capacity of DPPIV-positive hepatocytes to participate in repopulation of the biliary cell compartment.

Emerging evidence points to a stem cell niche in the hepatic stellate cells of the liver [212]. One lineage tracing study used GFP to label cells that expressed GFAP (a stellate cell marker) before subjecting mice to a diet-based model of liver injury and oval cell activation. After the injury, GFP-positive cells lost stellate cell markers and acquired stem/oval cell markers. These transitional cells disappeared as GFP-positive hepatocytes emerged [213]. 

## 4. Termination of Liver Regeneration

The processes governing the termination of liver regeneration are not as well studied or understood. Below we examine a few of the signaling pathways found to play a role in organ size regulation at the termination of liver regeneration.

### 4.1. Integrin-Linked Kinase (ILK)

As discussed earlier, one of the initiating events in regeneration after PHx is the remodeling of the extracellular matrix. Activation of metalloproteinases leads to the breakdown and release of proteins such as glysoaminoglycans, latent growth factors (HGF, TGF-β), and collagens. Later, when regeneration is nearing completion, new extracellular matrix is synthesized to re-establish the organ architecture. These processes are regulated by a fine balance of proteases (plasminogen activators, MMPs, plasmin) along with their inhibitors (TIMPs, PAI-1). Cells respond to the dynamic matrix turnover through integrins, which bind a variety of extracellular matrix components [214]. One intracellular mediator of integrin signaling is the integrin-linked kinase (ILK), which complexes with the proteins PINCH and parvin to mediate signaling [215]. 

The ILK signaling pathway serves to suppress hepatocyte growth. *In vitro*, hepatocytes cultured without extracellular matrix lose markers of hepatic differentiation and have enhanced proliferative capacity in response to HGF and EGF; when matrix preparations (such as Matrigel) are overlaid onto these cultures, the hepatocytes regain differentiation. ILK-deficient hepatocytes were not fully able to re-differentiate [216]. Hepatocyte-specific deletion of ILK in mice induces increased proliferation of hepatocytes and cholangiocytes for the first three months of life. Expression of genes associated with hepatocyte differentiation decreases over this time period. By three months of age, the mice have livers twice the size of normal, increased extracellular matrix accumulation around hepatocytes, altered matrix profile, and enhanced hepatocyte-associated genes (in contrast to earlier time points) [217]. When subjected to PHx, regeneration is prolonged and the final weight of the liver exceeds the original weight prior to surgery [31]. These observations highlight the essential role of extracellular matrix-mediated signaling in controlling liver size and suppression of proliferation at the termination of liver regeneration.

### 4.2. Glypican 3

Glypican 3 is a GPI-linked protein on the plasma membrane of hepatocytes that does not possess a signaling domain. It is one of the most highly expressed proteins in hepatocellular carcinoma and is measured clinically as a diagnostic marker [218]. It was thought that glypican 3 might be a growth promoter; however, loss-of-function mutations in humans (Simpson-Golabi-Behmel syndrome) are associated with organ enlargement [219], suggesting that it is a growth suppressor that is overexpressed in cancer but unable to halt the accelerated growth of the neoplastic cells. Studies in liver regeneration after PHx also support a growth-suppressing role of glypican 3. Expression of glypican 3 only begins at day 2 after PHx, reaching maximal levels at day 5. Suppression of glypican 3 in cultured rat hepatocytes enhances proliferation [220]. Further, overexpression of glypican 3 on hepatocytes in transgenic mice suppresses proliferation after PHx [32]. The mechanism behind these effects of glypican 3 is not clear, but one possibility is through modulation of the Hedgehog signaling pathway, which has been shown to be negatively regulated by glypican 3 during development [167].

### 4.3. TGF-β and Activins

As discussed above in section 2, TGF-β signaling is strongly mitoinhibitory for hepatocytes. It is purged from the liver at the initiation of regeneration after PHx but begins to be expressed in later stages. Re-establishment of local stores of TGF-β may represent one arm of the balance in holding hepatocytes in quiescence at the end of regeneration and in the resting liver. When a plasmid encoding a dominant negative TGF-β type II receptor is expressed in normal rat livers by adenovirus infection, DNA synthesis was significantly elevated starting at day 3 and peaking at day 7 after infusion [221]. No PHx was performed in this study, suggesting the increased proliferation may be a response to locally available growth factors (e.g., HGF) with the loss of tonic TGF-β signaling. TGF-β is bound to the matrix by decorin, a GPI-linked protein on hepatocytes, which has its own inhibitory effects on MET and EGFR function [222,223].

Activins are members of the TGF-β superfamily that have also been implicated in growth suppression in liver regeneration. Adenovirus injection of a dominant negative activin type II receptor also elevates hepatocellular DNA synthesis [221]. In addition, inhibition of activin using follistatin prolongs hepatocyte proliferation after PHx in mice, and this effect of follistatin is especially pronounced in mice lacking TGF-β type II receptors, which otherwise do not exhibit increased proliferation after PHx due to compensatory activin signaling [132].

### 4.4. Yes-Associated Protein (YAP)

YAP is the mammalian homologue of the Hippo kinase pathway target Yorkie (Yki) in Drosophila, a growth regulatory pathway [224]. YAP promotes the transcription of genes relating to cell differentiation and proliferation. When YAP is phosphorylated through activation of the kinase pathway (Mst1/2 in mammals), it is expelled from the nucleus and targeted for degradation. Overexpression of YAP in hepatocytes results in massive hepatic enlargement [225], and likewise genetic loss of Mst1/2 has similar consequences [226,227]. Compellingly, recent studies show that increased YAP nuclear localization correlates with perturbations causing liver enlargement [31,33,34], and likewise decreased nuclear YAP correlates with growth suppression [32,35]. Could YAP be the molecular “switch” that governs the hepatostat? More studies are needed to understand the signaling pathways that interact with and influence the activity of the Mst1/2 kinases and YAP localization, especially how this regulation connects to extracellular signals mediated by receptor tyrosine kinases and integrins. It is very likely, however, that this pathway is indeed a major component of the hepatostat.

## 5. Conclusions

The partial hepatectomy model has taught us that the process of liver regeneration is complex and intricate yet buffered by many redundant pathways. There is much evidence to suggest that liver regeneration in humans progresses similarly to rodents and many of the same growth factors and signaling pathways are relevant. These lessons learned from the PHx model have helped to inform clinical knowledge and strategies to bolster regeneration after surgical resection or transplant in patients [228,229], but also they serve as launching pads from which we can start to tease out the dysregulation of regeneration that occurs in chronic liver disease and cancer. 
